# Antibody to the Filarial Antigen Wb123 Reflects Reduced Transmission and Decreased Exposure in Children Born following Single Mass Drug Administration (MDA)

**DOI:** 10.1371/journal.pntd.0001940

**Published:** 2012-12-06

**Authors:** Cathy Steel, Joseph Kubofcik, Eric A. Ottesen, Thomas B. Nutman

**Affiliations:** 1 Laboratory of Parasitic Diseases, National Institutes of Health, Bethesda, Maryland, United States of America; 2 Lymphatic Filariasis Support Center, The Task Force for Global Health, Decatur, Georgia, United States of America; Michigan State University, United States of America

## Abstract

**Background:**

Antibody (Ab) to the *Wuchereria bancrofti* (Wb) infective larval (L3) antigen Wb123, using a Luciferase Immunoprecipitation System (LIPS) assay, has been shown to be a species-specific, early marker of infection developed for potential use as a surveillance tool following transmission interruption post mass drug administration. To examine its usefulness in a single filarial-endemic island assessed at two time points with markedly different levels of transmission, Ab to Wb123 was measured in sera collected from subjects from Mauke, Cook Islands in 1975 (no previous treatment) and 1992 (5 years after a one time island-wide treatment with diethylcarbamazine [DEC]).

**Findings:**

Between 1975 and 1992, Wb transmission decreased dramatically as evidenced by reduced prevalences of microfilariae (31% vs. 5%) and circulating Ag (CAg, 49% vs. 16%). Age specific prevalence analysis showed a dramatic reduction in Wb123 Ab positivity from 54% (25/46) in 1975 to 8% (3/38) in 1992 in children 1–5 years (p<0.0001), reflecting the effects of single-dose treatment five years earlier. By 1992, Wb123 Ab prevalence in children 6–10 years had fallen from 75% (42/56) in 1975 to 42% (33/79) consistent with a lower cumulative transmission potential. In the whole population, Wb123 seropositivity decreased from 86% to 60% between 1975 and 1992. In CAg+ subjects the levels of Wb123 Ab were indistinguishable between the 2 time points but differed in those who were CAg− (p<0.0001). In paired sample analysis, individuals who were CAg+ in 1975 but became CAg− in 1992 had significantly lower Ab levels in 1992 (p<0.0001), with 9/40 (23%) becoming seronegative for Wb123.

**Conclusions:**

The relationship between reduction in Wb123 Ab prevalence and the reduction of transmission, seen most clearly in young children, strongly advocates for the continuing assessment and rapid development of Wb123 as a surveillance tool to detect potential transmission of bancroftian filariasis in treated endemic areas.

## Author Summary

Lymphatic filariasis (LF) causes an enormous disease burden throughout the tropics and subtropics. The Global Programme to Eliminate Lymphatic Filariasis was begun in 2000 following the advent of large donations from drug companies for treating LF and the development of a rapid antigen assay for detection of infection. As more countries undergo mass drug administration (MDA), the driving need is for development of a highly sensitive and specific antibody assay for detecting ongoing exposure to vector-borne filaria following MDA. The target group for such surveillance is children born during or following MDA. Current assays, while sensitive, are not specific enough where non-LF filaria species are co-endemic. Recently, we developed an antibody assay based upon the highly specific larval antigen Wb123 using the Luciferase Immunoprecipitation System (LIPS). In the current study, we determined that the Wb123 LIPS assay detects a reduction in LF transmission on an endemic island following a one-time island wide MDA with diethylcarbamazine, with the most pronounced reduction in prevalence of antibody to Wb123 occurring in young children born just prior to and following this MDA. We propose that Wb123 can be an extremely useful surveillance tool following MDA and should be developed into a rapid test format.

## Introduction

The Global Programme to Eliminate Lymphatic Filariasis (GPELF) was begun in 2000 [1] in response to a World Health Assembly Resolution to eliminate filarial disease caused by *Wuchereria bancrofti, Brugia malayi* and *Brugia timori*. The success of this mass drug administration (MDA)-based program that utilizes the drugs diethylcarbamazine [DEC], ivermectin, and albendazole given as 2-drug combinations once-yearly, is evidenced by the more than 3.4 billion treatments given to nearly 897 million individuals in 53 of the 72 endemic countries during the first 10 years of the program [1]. The health [2] and economic [3] benefits of the GPELF have been well documented. This dramatic progress of the GPELF has been made possible by two key factors - first, the discovery of effective single-dose drug combinations that are able to markedly diminish microfilaremia (plus the subsequent donation of these drugs); and second, the development of a rapid diagnostic tool that detects active infection (the ICT circulating antigen [CAg] card test [4]) and enables rapid mapping of regions endemic for bancroftian filariasis.

As many of the first countries to implement MDA have already completed or are approaching completion of their elimination programs, the need for a sensitive and specific post-MDA surveillance tool has become a research priority [5], [6]. While the ICT card test provides the ability to measure the decrease in active infection rates following MDA, an antibody (Ab) based surveillance tool that detects early exposure in individuals would be preferable for determining whether or not filarial *transmission* has been successfully interrupted; the most effective sentinel group for such surveillance has been posited to be children born during or after the MDA [7], [8].

A number of serological Ab assays based on filarial Ags have been proposed for use as surveillance tools, including Bm14 [9], BmR1 and BmSXP [10], WbSXP-1 [11], and Bm33 [12]. The most widely field tested assays have used Bm14 Ag in ELISA [5], [8], [9], [13], [14] or other formats [7]. Results of an extensive multicenter evaluation of many of the available diagnostic assays (Ab, Ag, DNA) was recently reviewed (Gass, et al [15]). While sensitivity of the Ab assays has generally been high and specificity against non-filarial species has been relatively good, there are unresolved issues of specificity when tested against other filarial species, in particular, *Onchocerca volvulus* and *Loa loa* [5], [9] which are often sympatric with *W. bancrofti* (Wb) in Africa. In such co-endemic countries, the inability to distinguish individuals who are truly exposed to bancroftian filariasis from those who may be infected with or exposed to other filarial species could potentially confound the interpretation of surveillance findings post-MDA. In Wb endemic regions that lie within or in close proximity to *Loa*-, *Onchocerca*-, or *Mansonsella*-endemic areas the need for a highly specific Ab assay to detect *Wb* infection might well be essential to program success.

To address this need for a specific *W. bancrofti* diagnostic, a serological assay based on the *W. bancrofti* filarial antigen Wb123 was recently developed [Kubofcik et al.; in press, PLoS NTDs]. This antibody assay, utilizing the Luciferase Immunoprecipitation System (LIPS) technology [16], has been demonstrated to be not only highly sensitive in detecting Wb infections, but also highly specific, showing little to no cross-reactivity with sera from patients infected with filarial species other than Wb, including *O. volvulus*, *L. loa*, *Mansonella perstans*, and *B. malayi* [Kubofcik et al.; in press, PLoS NTDs]. However, since the relationship between this Wb123 Ab response and different population prevalence levels of infection or changes in these prevalence levels has not been defined, the current study was designed to take advantage of earlier, longitudinal serologic studies in a Pacific island population (Mauke, Cook Islands) to assess the effect of filarial transmission on serologic reactivity to Wb123 prior to and 5 years after a one-time, island-wide treatment with DEC [17]–[20].

## Materials and Methods

### Ethics Statement

Protocols for both population studies (1975 and 1992) on Mauke were approved by the government of the Cook Islands and the NIAID Institutional Review Board; informed written consent was obtained from all adult subjects. Consent from non-adult subjects was obtained through both verbal assent and written consent from each subject's legal guardian.

### Patient Population

The study population comprised the permanent residents of the island of Mauke in the Southern Cook Islands, a region endemic for the filarial parasite *W. bancrofti*; assessment occurred at two time points (1975 [n = 369; ∼58% of the population] and 1992 [n = 553; ∼88% of the population]; [17]–[20]). All subjects were evaluated for clinical (history, physical examination, and complete blood count), parasitologic (filtration of 1 ml of blood through a Nuclepore 3 mm filter to quantify microfilariae) and immunologic parameters during the time of both studies. Serum samples from both 1975 and 1992 were frozen in liquid nitrogen within hours of collection and then subsequently stored at −80°C. Sera were tested for the presence of both Ab and CAg (TropBioPty Ltd., Townsville, Australia). A summary of the assessments made on the individuals evaluated for the current Wb123 study is shown in Table 1.

**Table 1:** Patient population.

Age (years)	Number (M/F)*	# (%) CAg+	# (%) Mf+
**1975**			
<1–5	53 (21/32)	15/51 (29)	8/47 (17)
6–10	56 (28/28)	21/56 (38)	10/56 (18)
11–15	47 (19/28)	19/47 (40)	9/47 (19)
16–20	28 (8/20)	8/28 (29)	5/26 (19)
21–30	36 (15/21)	19/36 (53)	11/36 (31)
31–40	52 (27/25)	34/52 (65)	22/50 (44)
41–50	29 (14/15)	17/29 (59)	13/29 (45)
51–60	34 (15/19)	22/33 (67)	19/33 (58)
>60	34 (16/18)	19/32 (59)	13/32 (41)
**1992**			
<1	ND**	ND	ND
1–5	44 (31/13)	1/44 (2)	0/39 (0)
6–10	82 (44/38)	16/80 (20)	3/77 (4)
11–15	92 (56/36)	10/92 (11)	3/89 (3)
16–20	78 (34/44)	3/78 (4)	0/76 (0)
21–30	72 (34/38)	11/72 (15)	4/69 (6)
31–40	45 (23/22)	5/45 (11)	1/45 (2)
41–50	42 (24/18)	10/41 (24)	2/41 (5)
51–60	49 (24/25)	14/49 (29)	4/48 (8)
>60	49 (26/23)	18/49 (37)	7/46 (15)

* Number of patients relates to the number for whom Wb123 serology was done; the number of patients for whom Mf and CAg were done is shown in their respective columns.

** Not done.

### Luciferase Immunoprecipitation System (LIPS)

The LIPS assay for detection of IgG Abs to the Wb123 Ag is described in detail by Kubofcik et al. [in press, PLoS NTDs]. Briefly, 1 µl of sera was diluted 1∶10 in a Tris buffer in 96-well microtiter plates (Nunc, Roskilde, Denmark) and then added to 50 µl of a Ruc-Wb123 enzyme reporter. Plates were incubated for 5 minutes at room temperature after which 7 µl of a 30% suspension of protein A/G beads was added for another 5 minutes. Plates were washed and subsequently processed on a Berthold LB 960 Centro microplate luminometer using a colenterazine substrate mix (Promega, Madison, WI). Data were interpreted as luminometer units from averaged duplicate samples.

### IgG4 ELISA to Parasite Antigen

IgG4 Ab was measured by ELISA to a saline extract of adult *B. malayi* Ag (BmA) as described previously [21].

### Statistical Analyses

Statistical analyses were conducted using Graph Pad Prism (version 5.0). Geometric means (GM) were used to reflect central tendency. Comparisons of population parasitological parameters (i.e. microfilaremia [Mf] and CAg prevalence) and Wb123 positivity at the two study time points were carried out with the Fisher's Exact test. A comparison of Wb123 Ab levels between patients from the two time points was accomplished with the Mann-Whitney U test, while analysis of Ab levels in paired patients from 1975 and 1992 was carried out using the Wilcoxon Signed Rank test. Correlation analysis between IgG4 and Wb123 was accomplished with Spearman's Rank test.

## Results

### Population Dynamics and Wb123 Antibody Prevalence by Age

Transmission of *W. bancrofti*, although still ongoing in 1992, was reduced significantly between 1975 and 1992, most likely because of a one time island-wide treatment (MDA) of everyone ≥5 years old with diethylcarbamazine (DEC) in 1987, 5 years prior to the 1992 study [19]. Parasitological factors including Mf (number [%] positive = 111/360 [31%] in 1975 vs 26/560 [5%] in 1992) and CAg (number [%] positive = 178/360 [49%] in 1975 vs 88/558 [16%] in 1992) as well as Wb123 Ab positivity (319/369 [86%] in 1975 vs 334/553 [60%] in 1992) were all reduced in prevalence between 1975 and 1992 (p<0.0001 for each).

**Figure 1:**
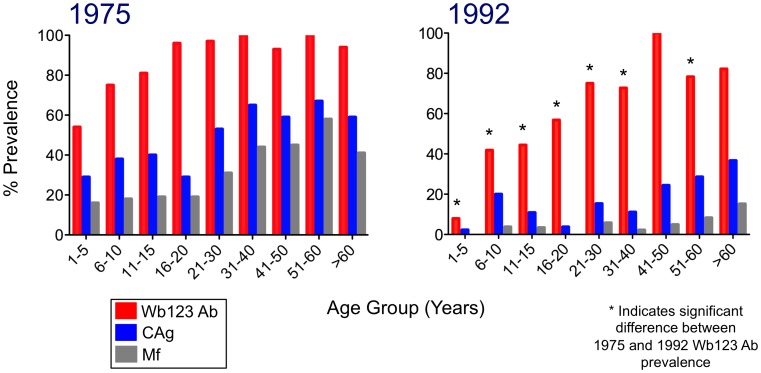
Prevalences of Ab to Wb123, CAg, and Mf in 1975 and 1992. Percent prevalence (y-axis) of microfilaremia (Mf; grey bars), circulating antigen (CAg; blue bars), and Ab to Wb123 (red bars) by age group (x-axis) for the study population on Mauke in 1975 (left panel) and 1992 (right panel). Asterisks represent a significant difference (p≤0.004; Fisher's exact test) between Wb123 Ab prevelances in 1975 and 1992 within a given age group.

The prevalence of Wb123 Ab by age groups, compared with that of Mf and CAg positivity, is illustrated in Figure 1. Wb123 positivity in 1975 and 1992 differed significantly (p≤0.003) at all but two age groups (31–40 years and >60 years). Indeed, the difference in Ab prevalence between the two observation points was greatest for children 1–5 years (positivity = 54% [25/46] in 1975 vs 8% [3/38] in 1992). In addition, age-specience curves were steeper and peaked earlier and higher in 1975 than in 1992, with 97% of those over 20 years being positive in the 1975 cohort but only 73% in 1992. In total, there was a decrease in Ab prevalence of 25%. CAg and Mf prevalences for all age groups were similarly much reduced in 1992 compared to 1975, again likely reflecting a major – but not complete – decrease in transmission in 1992. Not surprisingly, Ab prevalences decreased, but to a lesser degree than did the levels of Mf or CAg.

**Figure 2:**
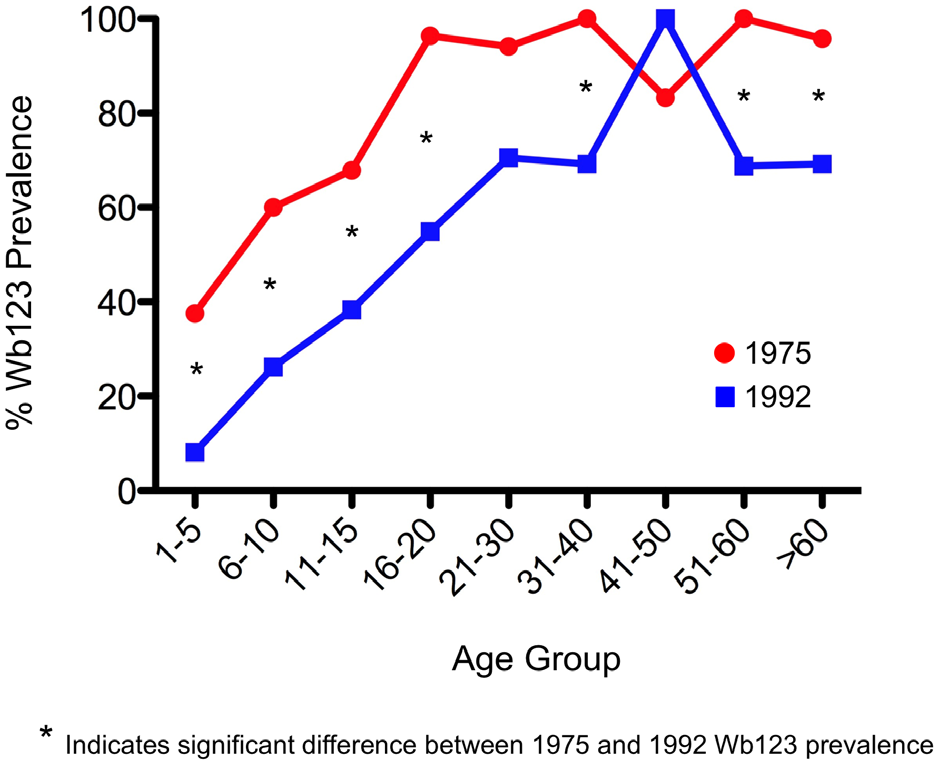
Prevalence of Ab to Wb123 in CAg− individuals. Percent prevalence (y-axis) of Ab to Wb123 by age group (x-axis) in CAg− individuals on Mauke from 1975 (red line) and 1992 (blue line). Asterisks represent a significant difference (p≤0.04; Fisher's exact test) between the prevelances in 1975 and 1992 within a given age group.

With but 2 exceptions, all CAg+ subjects in both 1975 and 1992 were also Wb123+ (data not shown). Therefore, to focus particularly on the Wb123 Ab levels in individuals who were *exposed* to Wb infected mosquitoes but who themselves were not actively infected, we evaluated the Ab prevalence in those individuals who were CAg− (Figure 2). Again, Wb123 prevalence was seen to be significantly higher in the 1975 population (exposed to greater Wb transmission) than in the 1992 population for nearly every age group. In comparing Wb123 Ab prevalences in CAg− individuals compared to the Wb123 Ab prevalence of the entire population (i.e. CAg+ and CAg−), the greatest difference was seen in children ≤15 years of age. By 16 years, the prevalence of Wb123 seropositivity was nearly identical in CAg− individuals compared to the entire population regardless of Ag status during both time periods (Figures 1 and 2).

### Quantifying Wb123 Antibody in 1975 and 1992

**Figure 3:**
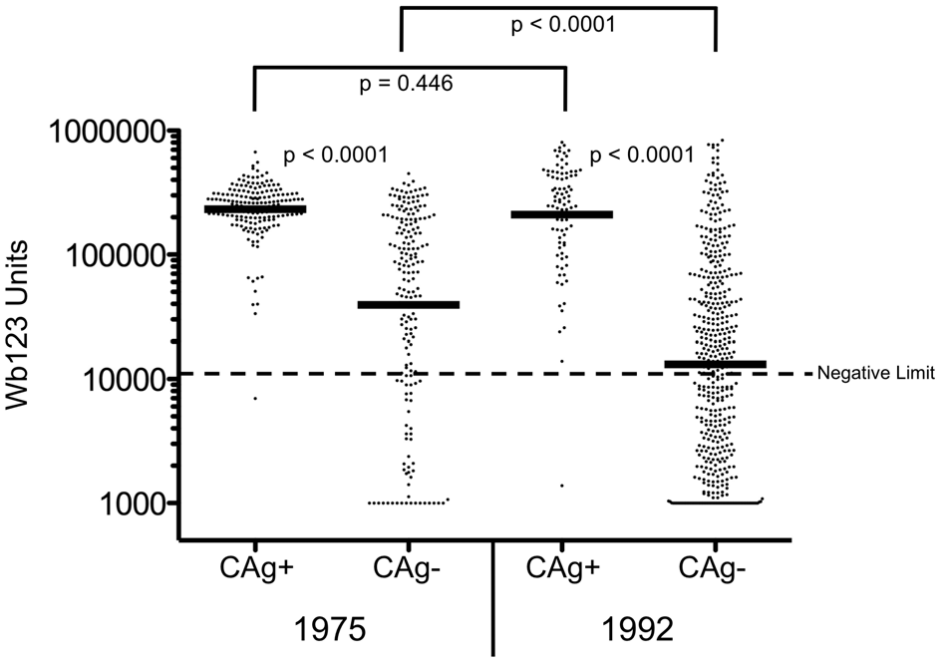
Levels of Ab to Wb123 by CAg Status in 1975 and 1992. Ab levels to Wb123 segregated by CAg status in subjects from 1975 (left side) and 1992 (right side). Each dot represents an individual patient, and the black bars represent the geometric means. The dashed line represents the lower limit of the assay (10,968 units) and p-values were calculated using the Mann-Whitney U test.

Figure 3 compares the level of Wb123 Ab in individuals as a function of CAg status. At both time points, Wb123 Ab levels were higher in CAg+ people than in those who were CAg− (geometric mean (GM) = 232,067 vs. 33,432 [1975] and 210,115 vs. 11,095 [1992]; p<0.0001 for both periods). Interestingly, the levels of Wb123 Ab in CAg+ patients did not differ between 1975 and 1992 despite the decrease in transmission; however, in CAg− subjects there *was* a significant decrease in Ab levels (p<0.0001) between 1975 and 1992. For both time points, the levels of Wb123 Ab and those of IgG4 Ab to BmA were strongly correlated (r = 0.671 [1975] and 0.729 [1992]; p<0.0001 for both time points) but there was no correlation between the levels of CAg and Wb123 (data not shown).

### Wb123 Antibody Production in Patients Evaluated at Both Time Points

**Figure 4:**
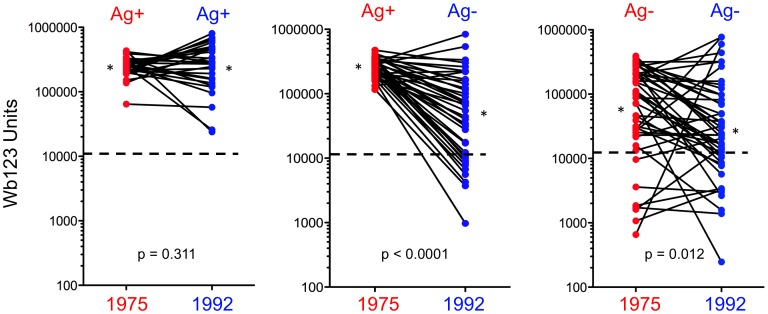
Levels of Ab to Wb123 in Patients Seen in Both 1975 and 1992. Change in the production of Ab to Wb123 as measured by LIPS in individuals seen in both 1975 (red dots) and 1992 (blue dots) segregated by CAg status. Each line represents an individual patient (n = 28 [CAg+ to CAg+]; n = 40 [CAg+ to CAg−]; n = 46 [CAg− to CAg−]); asterisks represent the geometric means for each group. The dashed line in each graph represents the upper limit of the normal level (10,968 units) of the LIPS assay and p-values were calculated using the Wilcoxon Signed Rank test.

A group of individual patients evaluated in both 1975 and 1992 [19] was studied to determine whether a change in CAg status affected Wb123 Ab production (Figure 4). Individuals who were CAg+ in 1975 and remained positive in 1992 (n = 28) showed no change in the level of Ab production to Wb123 with all patients remaining seropositive. However, those subjects who were either CAg+ in 1975 and became CAg− by 1992 (n = 40) or who were CAg− at both time points (n = 46) did show significant decreases in Wb123 Ab production in 1992 (p<0.0001 and p = 0.012 respectively) with 9/40 (23%) and 12/46 (26%) becoming or remaining seronegative respectively. There were no differences in the initial 1975 levels of Wb123 Ab in those Ag+ individuals who remained Ag+ and in those who became Ag− by 1992. Only one person seen at both time points became CAg+ in 1992 after being CAg− in 1975; this individual showed a 10-fold increase in Wb123 Ab units from 37,622 to 320,000 LU.

### Wb123 Antibody in Children

**Figure 5:**
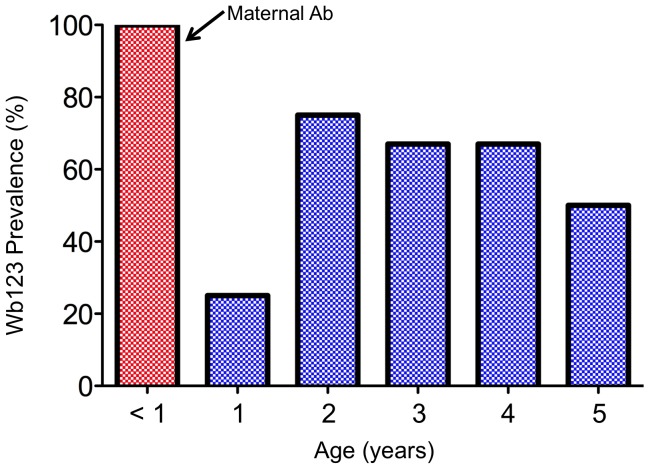
Prevalence of Ab to Wb123 in Young Children. Prevalence of Ab to Wb123 (i.e. the percent positive for Ab) in children ≤5 years old in 1975. The red bar for children <1 year presumably indicates the presence of maternal Ab.

To assess the effect of reduced transmission following MDA on Ab to Wb123 in young children (the target group for monitoring and evaluation currently recommended in WHO guidelines [22]), Ab levels were examined in more detail in this age group. Figure 5 shows the level of Wb123 Ab in young children ≤5 years in 1975 prior to any treatment. Interestingly, the occurrence of maternal Ab was quite evident in infants <1 year as 7/7 were positive for Ab. This prevalence dropped significantly (p = 0.003) in 1 year olds (3/12 [25%] Ab+) and then increased to levels of 50–75% in children 2–5 years of age.

**Figure 6:**
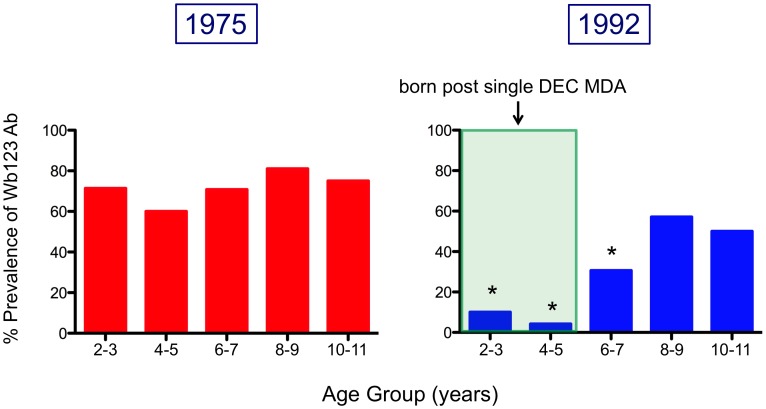
Prevalence of Ab to Wb123 in Children from 1975 and 1992. Prevalence of Ab to Wb123 (i.e. the percent positive for Ab) in children ≤11 years old by 2 year age divisions in 1975 (left panel; red bars) and 1992 (right panel; blue bars). The shaded green area represents the period of time where children were born after island-wide treatment with DEC. Asterisks indicate a significant difference (p≤0.0045) by Fisher's Exact test between identical age groups from both time periods.

Since children 6–7 years old will be the primary age group for monitoring post-MDA [22], Figure 6 illustrates the prevalence and levels of Wb123 Ab in children 2–11 years of age divided into 2-year age groups. Prevalence of Wb123 Ab in all children from 1975, prior to any treatment, remained high with a Wb123 Ab seropositivity of 60–81%. However, a steep and significant decrease in Ab prevalence was seen in children assessed in 1992 in the 2–3 (p = 0.0045), 4–5 (p = 0.0001), and 6–7 (p = 0.0035) year old groups compared with the same groups in 1975. Furthermore, it is these children (2–7 years) who were born either after or shortly before the single island-wide treatment with DEC. The prevalence of Wb123 Ab subsequently increases in older children and was not significantly different from that seen in 1975.

**Figure 7:**
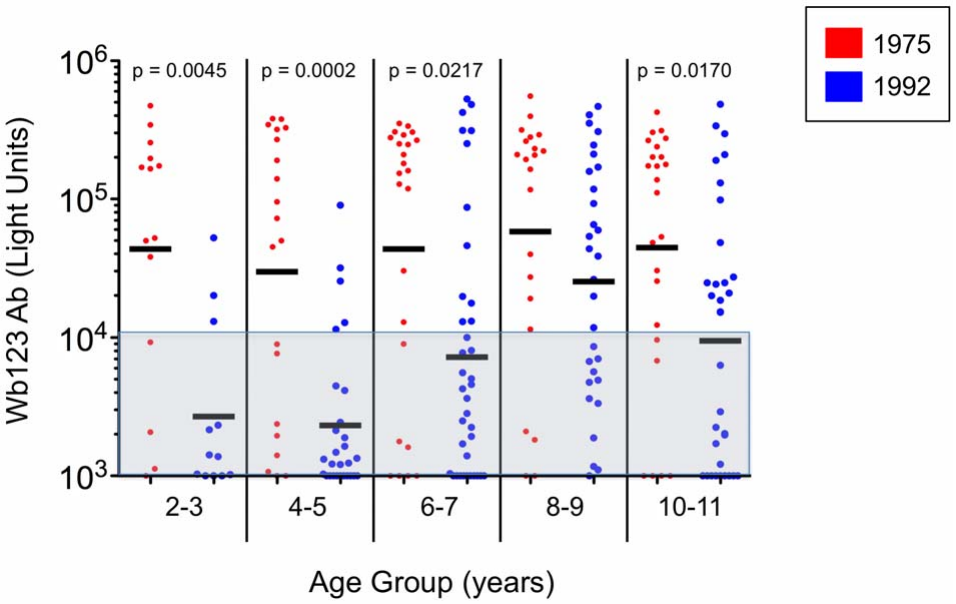
Levels of Ab to Wb123 in Children from 1975 and 1992. Ab levels to Wb123 in children ≤11 years old by 2 year age divisions in 1975 (red dots) and 1992 (blue dots). Each dot represents a single individual and the black bars represent the geometric means for each group. The shaded grey area indicates values considered negative and p-values were calculated using the Mann Whitney U test.

Comparison of Wb123 Ab levels in these children during the two time periods showed a similar pattern as prevalence (Figure 7). The level of Ab was significantly different in the age groups 2–3, 4–5, and 6–7 but not in 8–9 year olds. Interestingly, a significant decrease (p = 0.017) in Ab levels was seen in children 10–11 years old, possibly reflecting the beginning of reduced exposure to infected mosquitos seen in the population as a whole, particularly in those who were CAg− (Figure 3).

## Discussion

One of the greatest needs of the GPELF is for a highly sensitive and specific surveillance tool to monitor exposure to filarial infection in sentinel populations of young children born during or after MDA [7], [8] in regions completing the yearly MDA phase of their LF elimination programs [6], [7]. With MDAs nearing or having reached an end in several countries, the availability of such a surveillance tool has become even more urgent. Though highly specific circulating filarial antigen tests for detecting active infection [4], [8], [13], [14], [23]–[25] are well established (at least for Wb infection), a persistent challenge has been to devise an ‘exposure Ab’ test that is both sensitive and specific enough to be used in *W. bancrofti*-endemic countries that are also endemic for other filarial infections, particularly sympatric *Loa loa*, *Onchocerca volvulus* and *Mansonella* species. Indeed, many prior studies have identified highly sensitive Ab assays [5], [7]–[14], [25], [26], but none has had sufficient specificity to meet the current needs of the GPELF particularly in Africa and the Americas.

The Wb123 LIPs assay was developed to resolve this issue. This assay has proved both highly sensitive and specific – detecting exposure to *W. bancrofti* infection but not to other non-filarial helminth infections or to other filarial species [Kubofcik et al.; in press, PLoS NTDs]. The purpose of the current study was to test the performance of this new assay in a South Pacific island population assessed at two time points 18 years apart during which time the prevalence of *W. bancrofti* infection decreased dramatically. Included in this study were children born both before and after the single MDA.

The decrease in infection prevalence on the island of Mauke, defined by markedly lower CAg and Mf levels (Figure 1) in 1992, was accompanied by a significant decrease in the Wb123 Ab levels in essentially all population age groups, but the decrease was most marked in young children. A similar decline in Ab levels between 1975 and 1992 in this Cook Island population was seen for IgG, IgG4 and IgE antibodies to crude parasite Ag but only for the uninfected (‘endemic normals’) and those previously infected individuals who became CAg negative [19]. However, while CAg and Ab levels to Wb123 were not directly correlated in this study, previous findings did demonstrate that CAg and IgG4 levels to adult worm Ag were strongly correlated [27].

Some of the decreased Wb123 Ab response clearly reflects diminished levels of infection in the population, as one can infer from the longitudinal observations in individual patients (Figure 4) where those with persistent infection retained high Wb123 Ab responses but those who cleared their infections showed a dramatic fall in Ab levels. While this finding is interesting in itself, still more significant is the fact that when comparison of the Wb123 Ab rates at the two time points was restricted to those individuals who were CAg− (*i.e.,* presumably uninfected), the rates were uniformly lower across all ages groups in 1992 when the prevalence of infection in the population (and correspondingly the level of transmission) was much less than in 1975. Even in this CAg− population, Ab levels among the older population groups likely reflect not only current exposure to infection but also past exposure. However, in young children, Wb123 seropositivity appears to more closely reflect the actual exposure to infection (Figure 2), with the most pronounced differences seen in the youngest group of children (≤5 years) born post-treatment, a finding similar to that seen in other studies [14], [25]. Interestingly, the levels of Ab to Wb123 in the children 8–9 years of age did not differ between the two time periods whereas these levels differed significantly for all other age groups studied (Figure 7). The difference between those 8–9 year olds and the other groups might reflect their having been too young to receive the island-wide treatment but old enough to have been exposed to *W. bancrofti*, whereas the older group (10–11) would have received treatment, thereby lowering their Ab levels.

An additional finding in children was the presence of Wb123 Ab in all 7 children <1 year of age in 1975. Since Ab prevalence subsequently dropped to ∼20% in 1 year olds, this clearly indicates the presence of maternal Ab in this youngest group of children. There were not enough children <1 year old in 1992 to examine the effect of lower transmission, but presumably with fewer mothers infected the likelihood of a reduction in maternal Ab present in infants seems high.

The Wb123 LIPS assay gives every indication of being at least as effective as previously available assays for detecting anti-filarial Abs and, in fact, a comparison of Wb123 has been recently made with these other assays, including those assays for the detection of Abs to Bm33, Bm14 and WSP [Hamlin, et al.; in press, PLoS NTDs]. As evidenced in the present study and the report by Kubofcik et al. [in press, PLoS NTDs], the Wb123 assay combines three specific attributes that make it particularly valuable for use in the global LF elimination program: 1) it detects *exposure* to Wb infection; 2) its prevalence, especially in young children, reflects the Wb infection (and presumably transmission) levels in the population; and importantly 3) it is highly specific for detecting *W. bancrofti* infections, showing little or no cross-reactivity in sera from patients with onchocerciasis, loiasis, or mansonellosis.

Finally, the ability to detect exposure to infection in young children suggests that Wb123 will work effectively as a surveillance tool in this sentinel population. Indeed, as the GPELF increases the number of countries beginning and completing their MDAs, the development of the Wb123 assay into a rapid diagnostic would seem to be of enormous value both for the Global Programme's endgame and its post-MDA surveillance needs.
